# The anterior thalamic nuclei and cognition: A role beyond space?

**DOI:** 10.1016/j.neubiorev.2021.02.047

**Published:** 2021-07

**Authors:** Andrew J.D. Nelson

**Affiliations:** School of Psychology, Cardiff University, 70 Park Place, Cardiff, CF10 3AT, Wales, UK

**Keywords:** Thalamus, Hippocampus, Anterior cingulate cortex, Non-spatial learning, Context, Recency memory, Attention

## Abstract

The anterior thalamic nuclei are a vital node within hippocampal-diencephalic-cingulate circuits that support spatial learning and memory. Reflecting this interconnectivity, the overwhelming focus of research into the cognitive functions of the anterior thalamic nuclei has been spatial processing. However, there is increasing evidence that the functions of the anterior thalamic nuclei extend beyond the spatial realm. This work has highlighted how these nuclei are required for certain classes of temporal discrimination as well as their importance for processing other contextual information; revealing parallels with the non-spatial functions of the hippocampal formation. Yet further work has shown how the anterior thalamic nuclei may be important for other forms of non-spatial learning, including a critical role for these nuclei in attentional mechanisms. This evidence signals the need to reconsider the functions of the anterior thalamic within the framework of their wider connections with sites including the anterior cingulate cortex that subserve non-spatial functions.

## Highlights

• Anterior thalamic nuclei important for specific classes of temporal discriminations.
• Anterior thalamic nuclei required for hippocampal-dependent contextual processes.
• Critical role for anterior thalamic nuclei in selective attention.
• Significance of anterior thalamic – anterior cingulate interactions.

## 1 Introduction

Our understanding of the cognitive functions of the anterior thalamic nuclei has largely been framed by their connectivity with the hippocampus and other sites within the ‘extended hippocampal system’ including the retrosplenial cortex and mammillary bodies (Aggleton and Brown, 1999). An appreciation of this connectivity has motivated extensive research endeavours into the spatial functions of the anterior thalamic nuclei. The demonstration that pathology in the anterior thalamic nuclei is a critical component of diencephalic amnesia (Carlesimo et al., 2011; Harding et al., 2000; Segobin et al., 2019; Van der Werf et al., 2003) has similarly encouraged this focus on space, as aspects of spatial memory can be used to model episodic-like memory processes in animals (Aggleton and Pearce, 2001; Gaffan, 1991). A wealth of evidence has demonstrated how damage to the anterior thalamic nuclei produces profound deficits on an array of spatial tasks (Aggleton and Nelson, 2015; Jankowski et al., 2013; Wolff et al., 2015a). Descriptions of head-direction cells and other spatially responsive cells in the anterior thalamic nuclei have further underscored the importance of these nuclei for spatial memory and navigation (Jankowski et al., 2015; Matulewicz et al., 2019; Taube, 1995; Tsanov et al., 2011a).

Within the limbic thalamus, the functions of the anterior thalamic nuclei are often contrasted with those of the mediodorsal thalamic nucleus, the former important for space and the latter for tasks classically associated with frontal cortex (Mitchell, 2015; Wolff et al., 2015a). However, though understandable, the pre-eminence afforded to spatial memory in research into the functions of the anterior thalamic nuclei comes with potential risks. Indeed, there are good grounds to assume that the anterior thalamic nuclei may be implicated in cognitive processes beyond spatial learning and navigation. Diencephalic amnesia is not restricted to the spatial domain suggesting in the human brain, at least, that sites within the medial diencephalon including the anterior thalamic nuclei, ‘do’ more than just space (Aggleton and Brown, 1999; Carlesimo et al., 2011; Harding et al., 2000; Kopelman, 2015; Kopelman et al., 1997). Again consistent with a role in cognition beyond the spatial domain, evidence derived from *in vivo* intra-thalamic recordings in volunteer patients suffering from epilepsy has revealed correlates between anterior thalamic activity and successful verbal memory encoding (Sweeney-Reed et al., 2015, 2014), while P300-like potentials recorded in the anterior thalamic nuclei precedes activity in the hippocampus during the encoding of a visual memory task (Štillová et al., 2015). Further indirect support for this proposition comes from other neuropsychological findings of deficits in executive functioning and attentional processing following anterior thalamic nuclei damage (de Bourbon-Teles et al., 2014; Ghika-Schmid and Bogousslavsky, 2000; Lanna et al., 2012) as well as from evidence implicating the anterior thalamic radiation, consisting of fibres connecting the thalamus with the prefrontal cortex, in executive functioning (Biesbroek et al., 2013; Mamah et al., 2010). Furthermore, a consideration of anterior thalamic interconnectivity with sites beyond Papez circuit also points to the potential involvement of these thalamic nuclei in cognitive functions beyond the spatial domain. For example, the anterior thalamic nuclei are densely interconnected with frontal cortex (Mathiasen et al., 2017; Shibata, 1993a; Shibata and Naito, 2005; Wright et al., 2013). The functional importance of these non-hippocampal connections is, however, poorly understood.

Despite the overwhelming emphasis on spatial learning and navigation, there is a growing appreciation that the cognitive functions of the anterior thalamic nuclei are not limited to spatial learning and navigation. These advances in our understanding of the functions of the anterior thalamic nuclei will be the focus of this review. The behavioural analysis will largely be based on evidence derived from rodents, complemented, where possible, from studies involving humans. After briefly considering the connectivity of these nuclei, the role of the anterior thalamic nuclei in processing temporal and contextual information as well in learning and attention will be evaluated. A key consideration is this analysis is the extent to which these non-spatial functions mirror those of the hippocampal formation, and where they are related to potential interdependencies with sites other than the hippocampus.

## 2 Connectivity

The anterior thalamic nuclei are principally composed of the anteromedial, anteroventral, and anterodorsal nuclei. Although there is much overlap in terms of their connections, there are topographical and other differences, underscoring the need to consider each nucleus separately. Indeed, recent molecular work of the mouse thalamus has identified a striking pattern of transcriptional variation; with three distinct clusters of nuclei identified based on the topographical organisation of divergences in gene expression. Interestingly, none of the anterior thalamic nuclei colocalised on the same cluster (Phillips et al., 2019). This heterogeneity may in turn relate to functional specialisations.

### 2.1 Anterodorsal thalamic nucleus

**Fig. 1:**
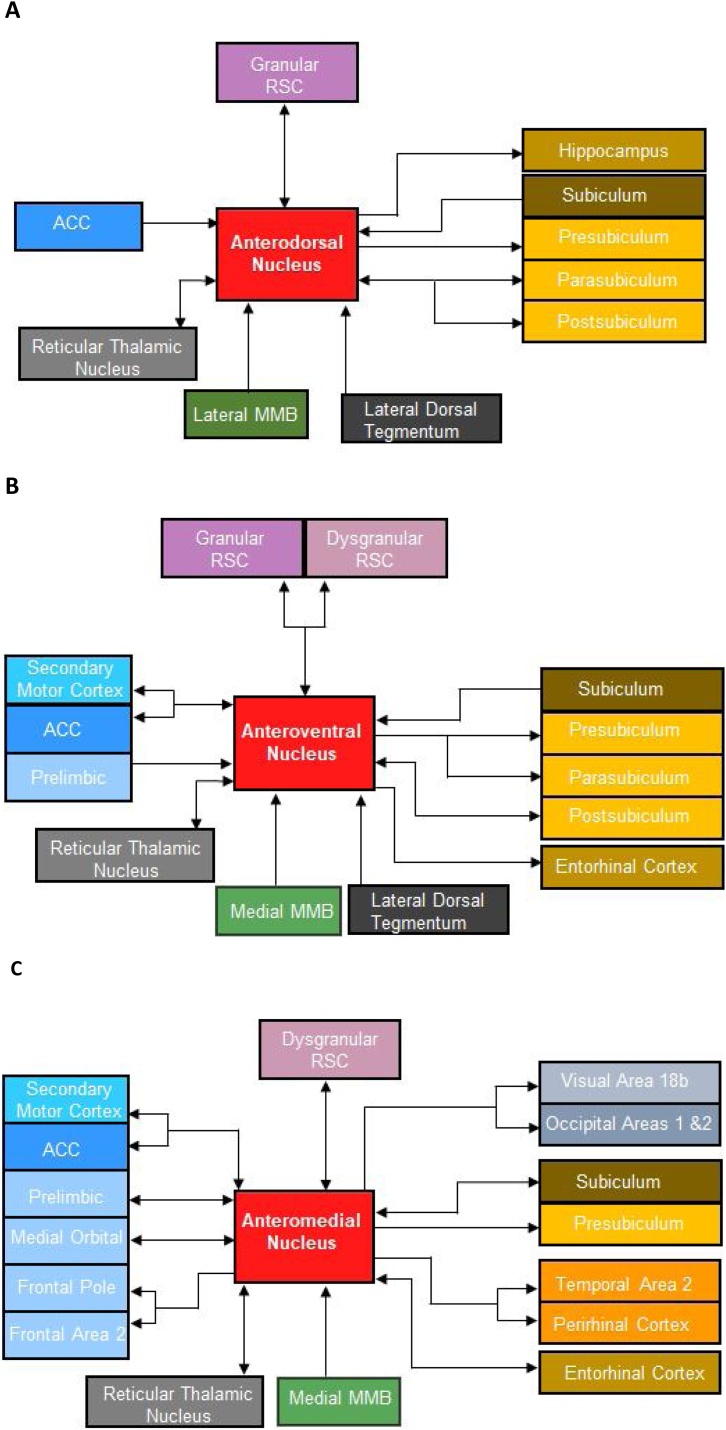
**Schematic detailing the principal anatomical connections of the anterodorsal (A), anteroventral (B) and anteromedial (C) thalamic nuclei.** Abbreviations: ACC, anterior cingulate cortex; MMB, mamillary bodies; RSC, retrosplenial cortex.

Of the three anterior thalamic nuclei, the anterodorsal nucleus has the most limited set of interconnections (Fig. 1A), perhaps reflecting its specialised role as a key node within the head direction system (Taube, 2007). Consistent with the importance of this nucleus for head direction information, the principal ascending projections to this nucleus arise in the lateral mammillary bodies (Blair et al., 1998; Shibata, 1992; Watanabe and Kawana, 1980). Further subcortical afferents originate in the caudal dorsal reticular nucleus (Shibata, 1992) as well cholinergic inputs from the lateral dorsal tegmental nucleus (Satoh and Fibiger, 1986). Hippocampal and parahippocampal afferents arise from the subiculum, para- and postsubiculum (Seki and Zyo, 1984; van Groen and Wyss, 1990a,b; Wright et al., 2010), while anterodorsal thalamic nucleus efferents target the pre-, para- and postsubiculum (van Groen and Wyss, 1990a,b; Van Groen and Wyss, 1995) as well as the hippocampus (Wyss et al., 1979). The anterodorsal thalamic nucleus is also reciprocally connected with granular retrosplenial cortex (Shibata, 1993a; van Groen and Wyss, 2003, 1990c; Wright et al., 2010). The only other cortical inputs are light afferents that originate in the caudal anterior cingulate cortex (Shibata and Naito, 2005).

### 2.2 Anteroventral thalamic nucleus

In contrast to the anterodorsal nucleus, the anteroventral nucleus has a somewhat wider set of connections (Fig. 1B), potentially consistent with a broader role in cognition. Like all three anterior thalamic nuclei, the anteroventral nucleus receives dense ascending projections from the mamillary bodies; these originate in the medial rather than the lateral mammillary nuclei (Watanabe and Kawana, 1980). The caudal dorsal reticular nucleus and lateral dorsal tegmental nucleus provide further subcortical afferents (Satoh and Fibiger, 1986; Shibata, 1992). Dense inputs from the hippocampal formation originate from the subiculum (Christiansen et al., 2016; Wright et al., 2010) as well as a lighter input from postsubiculum (van Groen and Wyss, 1990a). The projections back target pre-, para- and postsubiculum (Shibata, 1993b; van Groen and Wyss, 1990a; Van Groen and Wyss, 1995). The anteroventral nucleus is reciprocally connected with anterior cingulate cortex, both granular and dysgranular retrosplenial cortex and secondary motor cortex (Shibata, 1993a; Shibata and Naito, 2005; van Groen and Wyss, 2003, 1990c; Wright et al., 2010). Additional cortical efferents of the anteroventral nucleus terminate in entorhinal cortex (Shibata, 1993b). Finally, the anteroventral nucleus also receives afferents from prelimbic cortex (Wright et al., 2013).

### 2.3 Anteromedial thalamic nucleus

Of the three anterior thalamic nuclei, the anteromedial nucleus stands out by virtue of its connections with a wider array of cortical sites; with particularly dense connections with frontal areas (Fig. 1C), signalling their potential importance for non-spatial functions. Ascending projections originate in the medial mammillary bodies and rostral dorsal reticular nucleus (Shibata, 1992). Dense inputs from the hippocampal formation arise in the subiculum (Christiansen et al., 2016; Wright et al., 2010) while the anteromedial nucleus projects back to both the subiculum and presubiculum (Shibata, 1993b; van Groen et al., 1999). The anteromedial nucleus has dense reciprocal connections with anterior cingulate cortex and dysgranular retrosplenial cortex (Shibata, 1993a; Shibata and Kato, 1993; Shibata and Naito, 2005; van Groen et al., 1999; Wright et al., 2013, 2010). Further reciprocal connections with cortical sites include those with secondary motor cortices, medial orbital cortex, prelimbic cortex and entorhinal cortex (Mathiasen et al., 2017; Shibata, 1993b; Shibata and Kato, 1993; Shibata and Naito, 2005; van Groen et al., 1999; Wright et al., 2013, 2010). In addition, the anteromedial nucleus sends efferents to frontal area 2, frontal pole, visual area 18b, occipital area 1 and 2, temporal area 2 and perirhinal cortex (Shibata, 1993b; Shibata and Kato, 1993; van Groen et al., 1999).

## 3 Contextual processing

A cognitive domain closely associated with hippocampal function, is contextual memory (Bouton, 1993; Hirsh, 1974; Smith and Bulkin, 2014). Consequently, to test for parallels between hippocampal and anterior thalamic nuclei function, researchers have examined the impact of damage to the anterior thalamic nuclei on behavioural assays of contextual processing. It should be acknowledged that context is not a unitary construct and can mean different things depending on the nature of the task under investigation. Context is perhaps most often regarded as being synonymous with place, representing the background or the constellation of diffuse multimodal stimuli that are present when an event occurs. However, context extends beyond the physical attributes of the environment to include *intra alia* temporal information as well as internals states (Mizumori, 2013). Indeed, the binding together of these various elements are considered integral to episodic memory formation. At the same time, context can also be used to represent abstract rules or task-setting cues that are used by the animal to guide on-going behaviour, processes more closely associated with prefrontal function (Miller and Cohen, 2001).

### 3.1 Temporal information

Arguably one of the first demonstration that the anterior thalamic nuclei are involved in cognitive processes beyond the spatial domain came from investigations into the effect of anterior thalamic nuclei damage on behavioural tests that tax animals’ ability to discriminate between items based on the temporal order of events. This research builds on evidence that deficits in temporal memory are a consistent finding in neuropsychological assessments of patients with diencephalic pathology. Diencephalic amnesic patients are impaired on tests of recency memory that require judgments about the temporal context in which an item was encountered (Hildebrandt et al., 2001; Hunkin and Parkin, 1993; Kopelman et al., 1997; Parkin et al., 1990). However, given the diffuse nature of the pathology within the diencephalon in these patient groups as well as the presence of co-occurring frontal damage in Korsakoff’s patients, the neuroanatomical loci of these temporal order deficits remain unclear. Such considerations underscore the importance of comparative studies of this region.

It is now clear that anterior nuclei thalamic damage is sufficient to produce deficits on certain tasks that tax the use of temporal information. For example, excitotoxic lesions of the anterior thalamic nuclei impair rats’ ability to select an odour that was presented earlier in a list of odours in order to retrieve food rewards (Wolff et al., 2006). Importantly, the lesions spared recognition memory for individual items irrespective of their temporal relationships with other items, indicating that the deficit was related to an inability to use temporal information rather than a recognition memory deficit per se. Performance on this task is known to be hippocampal-dependent, with hippocampal lesions also selectively impairing rats’ ability to judge the position of two items from a recently encountered list of five odours (Fortin et al., 2002; Kesner et al., 2002).

Other work has exploited animals’ natural tendency to preferentially explore older familiar objects relative to more recently encountered familiar ones. This preference for older items can be used to index recency memory (Hannesson et al., 2004). Importantly, it has repeatedly been shown that the anterior thalamic nuclei are not required for the ability to discriminate on the basis of item familiarity *i.e*., standard tests of object recognition are not sensitive to anterior thalamic nuclei damage (Aggleton et al., 1995; Dumont and Aggleton, 2013; Mitchell and Dalrymple-Alford, 2005; Moran and Dalrymple-Alford, 2003). Consequently, any effect of anterior thalamic nuclei damage on tests of recency memory cannot not reflect an underlying deficit in recognition memory. It was initially reported that anterior thalamic nuclei lesions did not disrupt animals’ ability to discriminate between two objects presented in discrete temporal blocks (between block recency) (Mitchell and Dalrymple-Alford, 2005). Subsequent work has, however, found evidence that the anterior thalamic nuclei are required for certain classes of recency judgements (Dumont and Aggleton, 2013).

Using behavioural paradigms that involved rats discriminating between multiple objects encountered in discrete temporal blocks (between block recency) as well as multiple objects presented at different time points within a single continuous temporal block (within block recency), it was found that anterior thalamic lesions only impaired the latter class of recency judgements *i.e*. within block recency memory (Dumont and Aggleton, 2013). Potentially reflecting the dense interconnections between the anterior thalamic nuclei and retrosplenial cortex (van Groen et al., 1993; van Groen and Wyss, 1992, 1990c), the profile of spared between block but impaired within block recency mirrors precisely the performance of animals with retrosplenial cortex lesions on the same tasks (Powell et al., 2017). However, other work has shown that transection of the mammillothalamic tract, which disconnects the anterior thalamic nuclei from their dense mammillary bodies inputs, impaired both between and within block recency judgements using the same behavioural paradigm (Nelson and Vann, 2017). The greater impact of mammillothalamic tract lesions on this task may appear perplexing given that it is thought that almost all mammillary body cells project to the anterior thalamic nuclei (Takeuchi et al., 1985). A potential explanation is that the lesions in the Dumont and Aggleton study tended to spare the anteromedial nucleus, which sends projections to perirhinal cortex (Shibata, 1993b; van Groen et al., 1999) as well as being densely interconnected with prefrontal cortex (Shibata and Naito, 2005; Wright et al., 2013); sites both critical for recency memory (Barker et al., 2007).

Evidence from other behavioural tests that purport to tap temporal processes has been rather more equivocal. For example, anterior thalamic lesions do not disrupt rats’ ability to learn the temporal order of pairs of auditory and visual stimuli presented in an operant chamber (reinforcement occurs when stimulus A is presented before stimulus B, but not when stimulus B is presented before stimulus A) (Aggleton et al., 2011a). However, this temporal structural learning task involved multiple trials across multiple sessions. Anterior thalamic nuclei lesions impaired performance on a temporal alternation task in a delay-dependent manner (Beracochea et al., 1989; Célérier et al., 2000). However, this task again involved extensive training and the spatial aspects of the task further confounds interpretation of the findings. In contrast, those tasks where consistent deficits have been found involved one trial learning (Dumont and Aggleton, 2013; Wolff et al., 2006).

While the precise role of the anterior thalamic nuclei in recency memory may remain to be elucidated, it is, nevertheless, clear that, at least in rodents, their involvement is restricted to only the most challenging tests of recency memory. The role of the anterior thalamic nuclei in these processes is consequently dissociable from that of the mediodorsal thalamic nucleus, as damage to this region, like the prefrontal cortex with which it is reciprocally connected, disrupts even the simplest tests of between block recency memory (Aggleton et al., 2011b; Cross et al., 2012; Mitchell and Dalrymple-Alford, 2005). Similarly, hippocampal lesions disrupt between both between and within block recency tasks (Albasser et al., 2012; Barker and Warburton, 2011; Fortin et al., 2002; Kesner et al., 2002). These dissociations point to the potential existence of complementary pathways responsible for recency memory, with the role of the anterior thalamic nuclei restricted to situations when fine grained temporal discriminations between multiple items are required.

### 3.2 Context as ‘place’

Probably the most widely used task to assess contextual memory is contextual fear conditioning. To dissociate impaired contextual processing from a more general deficit in fear conditioning *per se*, it is important to show that cued fear conditioning is unaffected by the specific experimental manipulation. Importantly, most studies that have assessed the impact of anterior thalamic nuclei damage on fear memory have shown that cued fear conditioning remains intact after damage to the anterior thalamic nuclei (de Lima et al., 2016; Dupire et al., 2013; Ward-Robinson et al., 2002; but see Célérier et al., 2000). Pre-training anterior thalamic nuclei lesions appear to attenuate the acquisition of contextual but not cued fear conditioning, while at the same time leaving the expression of conditioned freezing intact when re-exposed to the conditioning context 24 h later (Dupire et al., 2013; Marchand et al., 2014). The same lesions also impaired remote retrieval of contextual, but not cued, fear memory assessed 3 weeks after conditioning (Marchand et al., 2014).

Consistent with these data, innate fear responses to a predator (a cat) are unaffected by combined anteromedial thalamic nucleus and nucleus reuniens lesions but the same lesions impaired fear responses to the context in which the predator was encountered (Carvalho-Netto et al., 2010). Whether these effects relate to impaired encoding or retrieval of contextual information is inevitably confounded by the permanent nature of the lesions used in this study. However, one study has reported increases in levels of the early immediate gene c-*fos* in the anterodorsal thalamic nuclei following the retrieval of a contextual fear response (Yasoshima et al., 2007). At the same time, subsequent work has used pharmacological inactivation (muscimol) to examine whether the involvement of the anterior thalamic nuclei in contextual fear conditioning is selective to specific stages of the procedure. These authors found that inactivating the anteromedial thalamic nucleus at encoding impaired contextual fear responses but inactivation prior to re-exposure to the context did not affect the expression or retrieval of contextual fear responses (de Lima et al., 2016), indicating that the anteromedial thalamic nucleus is required for the encoding but not the retrieval of contextual fear memories.

Dupire et al. (2013) also reported evidence that the involvement of the anterior thalamic nuclei in affective processes may extend beyond contextual fear memory. Rats with lesions in the anterior thalamic nuclei displayed reduced anxiety responses in the elevated plus maze, increased locomotor activity and reduced corticosterone responses when exploring a novel environment (Dupire et al., 2013). Whether the impairments in contextual fear conditioning are related to these decreases in emotional reactivity is not clear but there is no *a priori* reason to assume that such attenuated behavioural and physiological responses to anxiogenic stimuli would differentially affect contextual over cued fear conditioning. In any event, appetitive procedures obviate such concerns.

Importantly, there is evidence that appetitively motivated contextual tasks are sensitive to anterior thalamic nuclei damage. Transient inactivation of the anterior thalamic nuclei impaired animals’ ability to use contextual information to discriminate between different odours (Law and Smith, 2012). In this task, rats are trained on one list of odour discrimination problems, followed by training on a second list in either the same context or a different context. As the two lists contain overlapping items, control rats that learn the two lists in separate contexts outperform rats that learn the two lists in the same context. Inactivation of the anterior thalamic nuclei abolished this learning advantage, indicating that the use of contextual information to overcome interference requires the anterior thalamic nuclei (Law and Smith, 2012). A finding that reproduces the effects of temporary inactivation of the dorsal hippocampus on the same task (Butterly et al., 2012).

Dumont and Aggleton (2013) systematically investigated the performance of rats with anterior thalamic nuclei lesions on a series of contextual biconditional discriminations tasks (in context A, stimulus X is reinforced, Y is not reinforced; In context B, stimulus X is not reinforced, Y is reinforced). Consistent with the findings of Law and Smith (2012), anterior thalamic nuclei lesion animals were only impaired on tests of contextual processing that are sensitive to hippocampal damage. When contextual information was provided by the spatial arrangement of distal room cues, anterior thalamic nuclei lesions disrupted biconditional discrimination learning (Dumont et al., 2014), a profile of performance that mirrors the effects of hippocampal lesions on the same test of biconditional learning (Albasser et al., 2013). A finding that is of course not all surprising given that both the hippocampus and anterior thalamic nuclei are vital for processing allocentric information (Warburton et al., 2001). In contrast, anterior thalamic lesion animals readily acquired contextual biconditional tasks irrespective of whether contextual information was provided by different visual, thermal or floor texture cues (Dumont et al., 2014). Performance on biconditional discriminations of this nature are similarly unaffected by hippocampal lesions (Albasser et al., 2013; Coutureau et al., 2002). A potential explanation of this spared performance is that these contexts are composed of single elements rather than multimodal diffuse stimuli. Consequently, these tasks do not tax relational learning, whereby the animal must apprehend the interrelationship between multiple arbitrary stimuli. More broadly, these dissociations further highlight the need for precision when defining the nature of experimental contexts.

### 3.3 Contextual task-setting cues

While these aforementioned studies indicate that the anterior thalamic nuclei are engaged in contextual processes akin to those of the hippocampus, there is some preliminary evidence that the anterior thalamic nuclei may also be involved in the use of contextual information that represents abstract rules or task-relevant cues; functions more closely aligned with prefrontal cortex. In a behavioural paradigm that captures some of the response conflict features of the human Stroop task, rats concurrently learn two conditional discriminations, one visual and one auditory, in two distinct contexts (Haddon and Killcross, 2007, 2006). Each animal acquires four distinct instrumental contingencies. At test, animals receive compound audiovisual stimuli either composed of those stimulus elements that had elicited the same response (‘congruent’ trials) or different responses (‘incongruent’ trials) during training. Normal animals use contextual information to disambiguate the conflicting information provided by incongruent trials (Haddon and Killcross, 2007, 2006).

This task, therefore, assesses the use of higher order rules provided by contextual cues to guide instrumental behaviour. This ability depends on the integrity of prelimbic cortex (Haddon and Killcross, 2006; Marquis et al., 2007) as well as the retrosplenial cortex (Nelson et al., 2014). Anterior thalamic lesions selectively impaired performance during the initial (10 s) presentation of incongruent (*i.e*. conflicting) cues, but as incongruent trials progressed (remaining 50 s), the lesion animals were able to use contextual task-setting cues to disambiguate conflicting information and respond in a context-appropriate manner (Kinnavane et al., 2019). This profile of performance matches the effects of anterior cingulate cortex lesions on the same task (Haddon and Killcross, 2006) and is consistent with a role of these systems in the early detection of the conflicting task-relevant information. These findings provide a dissociation with the hippocampus, as hippocampal lesions facilitate rather than disrupt performance on this same task (Haddon and Killcross, 2007). A further implication is that some non-spatial functions of the anterior thalamic nuclei may be aligned with those of the anterior cingulate cortex (Kinnavane et al., 2019).

### 3.4 Interim summary

Taken together, the evidence from studies examining the role of the anterior thalamic nuclei in contextual processes suggests that their functions are broadly aligned with those of the hippocampus (Table 1). The finding that anterior thalamic damage impairs contextual conditioning when contextual cues are a proxy for place is entirely consistent with their known importance for processing allocentric information. The role of the anterior thalamic nuclei in temporal context is, however, far more limited than that of the hippocampus. The demonstration that anterior thalamic nuclei damage only impairs temporal discriminations involving multiple items encountered within the same temporal window, while sparing animals’ ability to discriminate between single items presented in discrete temporal blocks indicates that the involvement of the anterior thalamic nuclei is restricted to situations with high temporal interference. While other preliminary evidence suggests that the anterior thalamic nuclei may also play a role in the use of contextual cues as abstract rules that guide goal-directed behaviour; processes more closely associated with frontal areas.

**Table 1:** Summary of main findings from studies investigating the involvement of the rodent anterior thalamic nuclei in non-spatial processes.

Cognitive Domain	Task	Behavioural effects
Temporal context	Within Block Recency	✓*
	Between Block Recency	✗*
Context as ‘place’	Fear conditioning	✓*
	Biconditional discriminations - distal cues	✓*
	Biconditional discriminations - local cues	✗
Context as abstract rules	‘Stroop’ task	✓
Recognition memory	Novel object recognition	✗
Affective processes	Elevated plus maze	✓
Learning	Discrimination learning (electrophysiology)	✓
	(lesion studies)	✗
	Discrete cue conditioning (aversive)	✗
	Discrete cue conditioning (appetitive)	✗
	Reversal learning	✗
	Instrumental conditioning	✗
Attention	Vigilance/sustained attention	✗
	Intra-dimensional set-shifting	✓
	Extra-dimensional set-shifting	✓

✓ denotes behavioural effects have been found; ✗ denotes no behavioural effects; * denotes hippocampal-dependent task.

## 4 Learning and attention

### 4.1 Discrimination learning

Although the overwhelming focus of research into the functions of the anterior thalamic nuclei has in recent decades been on spatial learning and memory, there is, in fact, a venerable research history dating back to the 1970’s implicating the anterior thalamic nuclei in non-spatial learning. A body of evidence from work by Gabriel and colleagues indicates that the anterior thalamic nuclei are involved in learning processes that underpin both avoidance and appetitive discrimination learning. Unit recordings in the anterior cingulate cortex and anteroventral thalamic nucleus during an aversive avoidance task revealed that neuronal activity in both brain regions increased in response to presentations of a stimulus predictive of footshock (S+), while a control stimulus that was not behaviourally relevant (S-) did not evoke an equivalent neuronal response (Gabriel et al., 1977).

Subsequent work provided causal evidence to support this proposition. While lesions restricted to the anteroventral thalamic nucleus did not impair acquisition of the discrimination, the lesions disrupted extinction and reacquisition of the task. Furthermore, the lesions abolished discriminative neuronal firing in the anterior cingulate and retrosplenial cortices but not in the prefrontal cortex (Gabriel et al., 1983). A later study found that complete anterior thalamic nuclei lesions combined with mediodorsal thalamic lesions impaired acquisition of the avoidance task relative to both control and animals with lesions restricted to the mediodorsal thalamic nucleus. The lesions also abolished discriminative neuronal responses in the anterior cingulate and retrosplenial cortices (Gabriel et al., 1989). These findings have subsequently been replicated in an appetitively motivated version of the task (Smith et al., 2002).

Intriguingly, other work examining the impact of lesions in sites that innervate the anterior thalamic nuclei on avoidance performance as well as on discriminative neuronal responses, found dissociable effects of such manipulations. While mammillothalamic tract lesions impaired task performance and abolished the training-induced changes in neuronal firing within anteroventral thalamic, lesions in either the dorsal subiculum or retrosplenial cortex enhanced the discriminative discharges of anteroventral thalamic neurons (Gabriel et al., 1995; Gabriel and Sparenborg, 1986). These findings are consistent with the other evidence that mammillary body and hippocampal inputs make complementary, rather than overlapping, contributions to anterior thalamic function (Tsanov et al., 2011b; Wright et al., 2010).

Despite the wealth of evidence from this work, subsequent research has found little support for the involvement of the anterior thalamic nuclei in these processes, with far greater focus afforded to the role of the mediodorsal thalamic nucleus. Whether these discrepancies arise from species differences (rabbits *versus* rodents) or differences in task parameters (stimulus modality, response requirements, motivation) is not immediately clear. One critical factor might be that subsequent null results were found on tasks that generally require protracted training; potentially raising the possibility of functional compensation by other sites. In any event, evidence from a variety of behavioural paradigms has almost universally found that discrimination learning is unaffected by lesions in the anterior thalamic nuclei, with impairments only arising when the task places additional spatial demands on the animal. For example, anterior thalamic nuclei lesions do not disrupt visual discrimination tasks in a water tank, in which rats have to swim towards a visual stimulus that is associated with escape from the water irrespective of spatial location, while learning to ignore an alternative visual stimulus with no escape platform (Kinnavane et al., 2019; Moreau et al., 2013). Performance on appetitive conditional discriminations of this nature also appear insensitive to anterior thalamic nuclei damage (Chudasama et al., 2001; Kinnavane et al., 2019; Wolff et al., 2015b). Similarly, reversal learning assessed on these same tasks is unaffected by anterior thalamic nuclei damage (Chudasama et al., 2001; Kinnavane et al., 2019; Wolff et al., 2015b), whereas mediodorsal thalamic lesions can cause reversal deficits (Chudasama et al., 2001; but see Wolff et al., 2015b). Furthermore, anterior thalamic nuclei lesions do not impair animals’ ability to shift response strategies when contingencies change: lesion animals were able to switch as effectively as controls from a response to a visual strategy in an operant-based task, a task known to be sensitive to the effects of mediodorsal thalamic damage (Block et al., 2007; Kinnavane et al., 2019).

### 4.2 Instrumental learning

The evidence from these later studies would suggest that animals with anterior thalamic nuclei lesions are able to ascribe predictive value to discrete discriminative stimuli. The anterior thalamic nuclei also do not appear important for the control of instrumental responding, as anterior thalamic lesion animals are as sensitive to the causal consequences of instrumental actions as controls, and readily adapt behaviour in response to changes in reward value or degradation of the instrumental contingency (Corbit et al., 2003). One study that measured animals’ ability to differentiate between two arms of a T-maze after the reward associated with one of the arms had been devalued by pre-feeding the animals to satiety, found that anterior thalamic nuclei lesion animals were insensitive to the outcome devaluation procedure (Alcaraz et al., 2016). However, this effect most likely arises from a spatial memory deficit rather than an inability to update behaviour in response to changes in reward value. In contrast, the mediodorsal thalamic nucleus is important for representing goal value and the casual relationship between instrumental actions and their outcomes (Alcaraz et al., 2018; Corbit et al., 2003).

However, a recent report suggests that the anterior thalamic nuclei, and in particular the anteromedial thalamic nucleus, may have a hitherto unappreciated role in processes that support instrumental learning (Yang et al., 2020). These authors found that optogenetic stimulation of medial prefrontal cortex terminals within the anteromedial thalamic nucleus supported intracranial self-stimulation, with animals readily acquiring a lever press response to obtain photo-stimulation of these terminal fields. Interestingly, other thalamic targets of the medial prefrontal cortex only supported modest levels of intracranial self-stimulation (mediodorsal thalamic nucleus) or none at all (nucleus reuniens) (Yang et al., 2020). The same authors then confirmed that the reciprocal connections between the anteromedial thalamic nucleus and medial prefrontal cortex also support intracranial self-stimulation. Again, animals readily acquired a lever response to obtain photo-stimulation from optic-fibres targeting either neurons expressing channelrhodopsin within the anteromedial thalamic nucleus or their terminations in the medial prefrontal cortex (Yang et al., 2020). Consistent with the suggestion that these effects reflect reinforcement mechanisms, stimulation of these pathways in either direction resulted in activation of dopaminergic neurons in the ventral tegmental area (Yang et al., 2020). These results are preliminary and there is a clear need for further control experiments including an assessment of lever pressing for photo-stimulation in with animals not transfected with channelrhodopsin, the findings are, nonetheless, intriguing and raise questions about the role of the anteromedial thalamic nucleus and its interactions with prefrontal cortex in the control of goal-directed behaviour.

### 4.3 Attention

Although anterior thalamic nuclei lesions have no apparent impact on behavioural tests such as the five-choice serial reaction task that tax vigilance and sustained attention (Chudasama and Muir, 2001), recent evidence has revealed a critical role for the anterior thalamic nuclei in selective attention. Wright et al. (2015) tested two separate cohorts of rats with lesions in the anterior thalamic nuclei on an attentional set-shifting paradigm that captures two potentially conflicting attentional processes. In this task, normal animals show accelerated learning over successive discriminations solved by attending to a common stimulus dimension (intradimensional-set) (Mackintosh, 1975). This learnt bias to a specific stimulus class (attentional set) is further revealed when the stimulus dimension being rewarded switches to a qualitatively different category that has previously been irrelevant. Now, additional trials are required to solve this extradimensional shift, representing a ‘shift-cost’ (Birrell and Brown, 2000; Chase et al., 2012; Lindgren et al., 2013). It has been repeatedly shown that the ability to disengage from a previously rewarded response depends on the integrity of the prefrontal cortex, with damage to this region leading to response perseveration and a greater shift-cost (Birrell et al., 2000; Dias et al., 1996a,b). Given the dense interconnectivity of the anterior thalamic nuclei with some frontal sites, a key empirical question was whether lesions in the anterior thalamic nuclei would reproduce the known effects of prefrontal manipulations on this attentional task.

In a striking dissociation with the effects of prefrontal damage, anterior thalamic nuclei lesions disrupted animals’ ability to form an attentional-set as they were slower to learn discriminations involving a stimulus dimension that was consistently associated with reward (intradimensional shift) but, paradoxically, when required to solve a discrimination involving a hitherto irrelevant stimulus dimension (extradimensional-shift), the same animals outperformed control animals, displaying a shift-benefit (Wright et al., 2015). The generality of these findings was confirmed by a further set-shifting facilitation found for another stimulus class, spatial position. The implication of this unique learning profile is that the anterior thalamic nuclei are vital for attending to reliable predictors of reinforcement, driving attentional-set formation at the expense of extradimensional-shifts. In their absence, unreliable predictors of reinforcement usurp attentional control impairing intradimensional set-shifting but facilitating extradimensional shifts (Pearce and Hall, 1980; Pearce and Mackintosh, 2010).

Given that this profile of performance is diametrically opposed to the effects of prefrontal damage on this task (Birrell et al., 2000), this raises the question of which sites do the anterior thalamic nuclei interact to support these attentional mechanisms. Subsequent work has shown that chemogenetic inhibition of the anterior cingulate cortex reproduces exactly the effects of anterior thalamic nuclei lesions on this task *i.e*., impaired attentional set-formation but facilitated extradimensional set-shifting (Bubb et al., 2020). Further work using DREADDs to selectively disrupt anterior cingulate terminals in the anteroventral and anteromedial thalamic nuclei has confirmed that interactions between these two sites are required for animals to attend to reliable predictors of reinforcement (Bubb et al., 2020). Taken together these findings not only reveal that the anterior thalamic nuclei have vital attentional functions, but they also highlight the importance of their interconnections with sites beyond Papez circuit. In many respects, these findings were foreshadowed by Gabriel and colleagues work identifying neurons in the anteroventral thalamic nucleus that preferentially increased their firing in response to task-relevant cues. This same work also highlighted the significance of interactions with anterior cingulate cortex in these processes (Gabriel et al., 1977).

Importantly, the findings of Wright et al., 2015 have been corroborated by evidence from a study of attentional processes in human patients with lesions in the anterior and ventrolateral thalamus (De Bourbon-Teles et al., 2014). Healthy controls and patients completed a compound visual search task in which participants had to detect a target among distractors. The targets were composed of two features (shape and colour) with colour the distinguishing feature. At the beginning of each trial, participants were presented with a cue that matched the target colour (valid cue) or the distractor’s colour (invalid cue). In healthy participants, valid cues led to faster reaction times consistent with an attentional bias towards the relevant stimulus feature. In contrast, the patient group did not show this validity effect, with some patients showing the reverse effect of faster reaction times on invalid cue trials (De Bourbon-Teles et al., 2014). These findings therefore mirror those of Wright et al. (2015), with damage to the anterior thalamic nuclei disrupting attention to relevant cues while facilitating performance involving irrelevant information (Leszczyński and Staudigl, 2016). Of course, the usual caveats about evidence derived from human neuropsychological studies apply, as the damage in this patient group may have additionally involved fibres of passage resulting in thalamo-cortical disconnections (*e.g*., Nishio et al., 2014). However, the conclusion was supported by a subsequent fMRI study in healthy participants which showed that the fMRI signal in the anterior thalamic nuclei increased linearly as participants learned that cues predicted the target features (De Bourbon-Teles et al., 2014).

The concordance between the findings from studies involving rodents and human participants on the role of the anterior thalamic nuclei in attentional processes is particularly striking. The implication from both studies is that the anterior thalamic nuclei guide attention towards stimuli that reliably predict important outcomes. This evidence inevitably opens up many new research questions about the wider role of the anterior thalamic nuclei in cognition. Are such effects detectable on other learning tasks? If this attentional role represents an overarching property of the anterior thalamic nuclei, do such effects contribute to deficits seen on seemingly unrelated tasks?

Of particular interest is how these attentional effects relate to the more established role of the anterior thalamic nuclei in mnemonic processes. As interactions between attention and memory are required for successful memory formation (Aly and Turk-Browne, 2016; Chun and Turk-Browne, 2007; Fernandes et al., 2005; Muzzio et al., 2009), one potential implication of these findings is that the anterior thalamic nuclei may act as an attentional hub gating information flow to support memory encoding (Leszczyński and Staudigl, 2016). Of note are findings from a recent fMRI study showing that deactivation of the anterior thalamic nuclei during encoding of a face-scene associative memory task was related to subsequent recall (Geier et al., 2020). A finding that the authors interpret as evidence that the anterior thalamic nuclei contribute to the gating of irrelevant information during memory encoding (Geier et al., 2020). A conclusion that is broadly consistent with human electrophysiological evidence that cross-frequency coupling between theta and fast oscillations observed in the anterior thalamic nuclei during rest is attenuated when participants engaged in tasks involving an external focus of attention (Sweeney-Reed et al., 2017). Both these findings indicate that the anterior thalamic nuclei may be involved in process whereby relevant information is selected for subsequent encoding.

A clear priority for future work will be to determine how these attentional functions interact with hippocampal-mnemonic processes. One possibility is that the anterior thalamic nuclei modulate information flow between the hippocampus and prefrontal cortex facilitating the allocation of attention to relevant stimuli to aid memory formation. Given the dense interconnections between the anteroventral and anteromedial thalamic nuclei with both the hippocampal formation and frontal cortices (Fig. 1B/C), these thalamic nuclei are anatomically well-placed to subserve such a function. Such a modulatory role may relate to the synchrony of theta oscillations (4−8 Hz) by the anterior thalamic nuclei (Ketz et al., 2015). The coordination of theta rhythms between the hippocampus and frontal cortices is known to be involved in learning and memory in both animals and humans (Anderson et al., 2010; Benchenane et al., 2010; Jones and Wilson, 2005; Nyhus and Curran, 2010). Significantly, cells throughout the anterior thalamic nuclei file rhythmically in synchrony with hippocampal theta oscillations (Albo et al., 2003; Vertes et al., 2001), thereby providing a potential mechanism for such a coordinating role. Interestingly, electrophysiological recordings in epilepsy patients have demonstrated a relationship between neocortical-anterior thalamic theta synchrony and successful memory formation (Sweeney-Reed et al., 2015, 2014), while further work has shown how theta oscillations in the anterior thalamic nuclei and nucleus accumbens aid memory retrieval (Bauch et al., 2018).

## 5 Summary and conclusions

While the pre-eminence afforded to spatial cognition in research into the cognitive functions of the anterior thalamic nuclei is understandable, the evidence reviewed here indicates that the focus needs to be widened to consider non-spatial attributes. Some of this evidence suggests that the parallels between the anterior thalamic nuclei and the hippocampus apply equally to non-spatial functions. Indeed, evidence for the importance of the anterior thalamic nuclei for hippocampal-dependent contextual and temporal processes fits with the proposition that interdependencies between these sites are critical for aspects of both spatial and non-spatial cognition (Table 1). However, other data signal the need to consider anterior thalamic interactions with sites beyond the hippocampus. In particular, the emerging picture that the anterior thalamic nuclei have a vital role in modulating attention to reliable predictors of biologically significant events coupled with other evidence implicating these nuclei in further aspects of learning, points to the significance of interdependencies with anterior cingulate cortex (Table 1). Yet, we are only just beginning to appreciate the potential importance of these circuits.

Furthermore, these considerations highlight the need to address the unique contribution of each of the anterior thalamic nuclei to cognition. There is strong evidence to suggest that the anterodorsal nucleus, by virtue of its position as a key node within the head-direction system, has a specialised role in spatial learning and navigation (Jankowski et al., 2013; Taube, 2007). However, the sometimes-myopic focus on the anterodorsal thalamic head direction navigational system has the potential to overshadow the differential contribution of the anteromedial and anteroventral thalamic nuclei to cognition. Indeed, while the loss of the anterodorsal head direction signal can disrupt the stability of other spatial representations thought to support memory (place and grid cells) (Calton et al., 2003; Winter et al., 2015), these effects are not sufficient to account for anterior thalamic involvement in cognition as lesions in the head-direction system only produce mild or transient deficits on tests of spatial memory (Dillingham and Vann, 2019; Vann, 2018, 2005). However, the evidence to dissociate the contributions of the anteromedial and anteroventral nuclei is currently lacking. The recent demonstration of marked divergences in transcriptional factors between the three anterior thalamic nuclei further underscores the need to examine their separate contributions to cognitive functions (Phillips et al., 2019).

Consideration of the differences in the profile of connectivity of the three nuclei provides an important road map to uncovering their specific contributions to cognition. Based on its dense interconnections with the hippocampal formation and parahippocampal sites (Fig. 1B), it is highly likely that the anteroventral nucleus plays a key role in hippocampal-dependent spatial and non-spatial memory processes. In contrast, the anteromedial nucleus shares connections with an array of sites beyond the hippocampus and parahippocampal areas (Fig. 1C). Perhaps of particular note are its dense connections with frontal areas suggesting that the anteromedial nucleus may serves as a site of integration between frontal areas and the hippocampus to modulate attentional and cognitive control processes that underpin mnemonic functions. The recent evidence that inhibiting information flow from the anterior cingulate cortex to the anteromedial thalamus is sufficient to disrupt attention to task-relevant information is consistent with this proposition (Bubb et al., 2020). At the same time, anteromedial interconnections with the lateral entorhinal and perirhinal cortices, sites important for object information, suggest that the anteromedial nucleus may similarly act as an interface between frontal and hippocampal/parahippocampal areas that contribute to recency memory and other forms of associative recognition memory.

There remain many outstanding questions. Inevitably, some of the challenges to this endeavour are technical. However, the increasing availability of chemogenetic and optogenetic approaches that allow researchers to manipulate experimentally specific pathways should greatly advance our ability to dissect the differential involvement of each of the anterior thalamic nuclei to cognition. As ever, clearly defined behavioural paradigms and analysis are critical to forming a complete picture. While the electrophysiological properties of the anterodorsal nucleus have received considerable attention, characterizing the electrophysiological properties of the anteroventral and anteromedial nuclei requires further work. For example, it has long been recognised that cells within the anterior thalamic nuclei are modulated by theta (Albo et al., 2003; Vertes et al., 2001), but how this modulation contributes to function is not understood. The increasing application of multichannel recordings across multiple brain sites should allow researchers to assess whether this theta-modulation aids coordination between cortex and hippocampus and how this relates to function (Ketz et al., 2015). There is already electrophysiological evidence from human studies that synchronisation between the anterior thalamic nuclei and neocortex as well between the anterior thalamic nuclei and nucleus accumbens supports memory (Bauch et al., 2018; Sweeney-Reed et al., 2015, 2014) but work in animals should provide greater anatomical specificity as well as the ability to record concurrent activity across multiple brain sites. Disentangling the proposed attentional functions of the anterior thalamic nuclei from their role in memory will also require electrophysiological approaches. Outstanding questions include whether the anterior thalamic nuclei actively modulate attentional information or whether their attentional properties relate to the modulation of information flow between cortex and the hippocampus to support memory formation (Leszczyński and Staudigl, 2016).

Of course, the ultimate goal of comparative approaches is to understand the human brain. The translation between animal and human work is often challenging; particularly in the context of the anterior thalamic nuclei given their location and size in the human brain. However, recent human work and the apparent concordance between findings from human and animal studies is encouraging (De Bourbon-Teles et al., 2014; Geier et al., 2020; Leszczyński and Staudigl, 2016; Sweeney-Reed et al., 2017, 2015, 2014), raising the prospect of greater synthesis of the two approaches in the future.

These considerations inevitably feed into wider debates about the role of the thalamus in cognition and the need to supplant outdated views of the thalamus as a mere relay station to cortex (Mitchell et al., 2014; Perry and Mitchell, 2019; Sherman, 2007; Wolff and Vann, 2019). It is now recognised that pathology in the anterior thalamic nuclei is a core feature of diencephalic amnesia (*e.g*., Harding et al., 2000) but the anterior thalamic nuclei have also been implicated in an array of different neurological conditions that present with cognitive disturbances (Aggleton et al., 2016; Perry et al., 2019; Young et al., 2000); further reinforcing the need to better understand their unique contributions to cognition.

## Declaration of Competing Interest

The authors report no declarations of interest.
